# Anti-Osteoporotic Effects of Commiphora Myrrha and Its Poly-Saccharide via Osteoclastogenesis Inhibition

**DOI:** 10.3390/plants10050945

**Published:** 2021-05-10

**Authors:** Youn-Hwan Hwang, Ami Lee, Taesoo Kim, Seon-A Jang, Hyunil Ha

**Affiliations:** 1 Herbal Medicine Research Division, Korea Institute of Oriental Medicine (KIOM), 1672 Yuseong-daero, Yuseong-gu, Daejeon 34054, Korea; hyhhwang@kiom.re.kr (Y.-H.H.); dmb01367@kiom.re.kr (A.L.); xotn91@kiom.re.kr (T.K.); white7068@kiom.re.kr (S.-A.J.); 2 Korean Convergence Medicine Major KIOM, Korea University of Science & Technology (UST), 1672 Yuseongdae-ro, Yuseong-gu, Daejeon 34054, Korea

**Keywords:** myrrh, polysaccharide, osteoporosis, osteoclastogenesis, mice

## Abstract

In traditional oriental medicines, *Commiphora myrrha* and its resinous exudate (i.e., myrrh) are used as herbal remedies to treat various inflammatory and metabolic disorders. Until now, *C. myrrha*-derived herbal products are considered useful source for bioactive compounds to manage numerous human diseases. This study investigated the effects of water extract of *C. myrrha* resin (WCM) and its polysaccharide (WCM-PE) on modulatory effects of osteoclast differentiation and/or ovariectomized-induced bone loss. Oral administration of WCM (200 and 500 mg/kg/day for four weeks) notably decreased trabecular bone loss and lipid accumulation in the bone marrow cavity. WCM and WCM-PE dose-dependently inhibited receptor activator of nuclear factor-κB ligand (RANKL)-induced osteoclastogenesis and suppressed RANKL-mediated overexpression of c-Fos and nuclear factor of activated T cells, cytoplasmic 1, thereby downregulating osteoclast-specific gene (Atp6v0d2, DC-STAMP and cathepsin K) expression. Thus, our results suggest that WCM and WCM-PE are promising nutraceutical candidates for the management of osteoporosis in postmenopausal women.

## 1. Introduction

Osteoporosis is an abnormal bone remodeling disease characterized by low bone density and microarchitectural disruption that poses a threat to the elderly as well as menopausal women with excessive osteoclast activation and bone resorption [1]. During menopause, estrogen deprivation rapidly causes bone loss and porosity via an increase in osteoclast activation, because estrogen is closely implicated in bone turnover and homeostasis [2]. Osteoclasts derived from monocyte/macrophage lineage cells are unique multinucleated bone-resorbing cells that secrete tartrate-resistant acid phosphatase (TRAP) and proteases [3]. Osteoclast differentiation is initiated by receptor activation of nuclear factor-κB ligand (RANKL) and the nuclear translocation of c-Fos and nuclear factor of activated T cells, cytoplasmic 1 (NFATc1) following RANKL stimulation triggers osteoclast-specific gene expression (e.g., TRAP, cathepsin K, dendritic cell-specific transmembrane proteins (DC-STAMP) and ATPase H+ transporting V0 subunit D2 (Atp6v0d2)) [4]. Finally, the increased formation and maturation of osteoclasts results in bone resorption and loss. In this context, modulation of osteoclast differentiation and its resorptive function by bisphosphonates, denosumab and RANKL antibodies has become a feasible approach to treat patients with osteoporosis [5]. Although these conventional therapies efficiently protect against bone loss and fractures, they have been found to cause undesirable effects, such as gastrointestinal intolerance and a risk of jaw necrosis, indicating the limitations of their long-term use [6]. Thus, development of alternative treatment strategies, such as nutraceutic and therapeutic agents derived from natural sources, have been attracting attention considering the therapeutic and prophylactic benefits of these plant-derived products on bone health [7].

In several decades, various researchers provide beneficial effects of herbal medicines in post-menopausal management and bone health [8,9]. Bioactive compounds derived from medicinal plants in traditional oriental medicine have found to have anti-reabsorptive or anabolic properties in bone remodeling [10]. Indeed, treatment of soy isoflavones as an alternative therapy to hormone replacement therapy showed elevation of bone mineral density and inhibition of the bone resorption in postmenopausal women [11]. One of promising herbal medicines with alternative potential is that derived from *Commiphora myrrha* (Nees) Engl.

*C. myrrha* (family Burseraceae) is a small thorny tree that is widely distributed in Africa, Arabia and Asia [12]. *C. myrrha* and its derived products has been used to treat infection, inflammatory diseases, obesity, fracture and arthritis [13]. Particularly, the resinous exudate of *C. myrrha* (i.e., myrrh) in traditional oriental medicine has been used to improve wound care, joint pain, gastric dysfunction and burns [13]. The chemical composition of myrrh includes water-soluble gum, alcohol-soluble resin and volatile oil [14]. The gum of myrrh consists of polysaccharides and proteins, whereas the volatile oil contains steroids, sterols and terpenes. In the USA, myrrh has been approved as a safe natural flavoring and seasoning agent and preservative in foods and beverages by the Food and Drug Administration [15]. Recently, myrrh-derived products have gained increasing interest as therapeutics and dietary supplements owing to their various beneficial properties, including antiviral, anti-obesity, anti-cancer, hepatoprotective and hypoglycemic activities [15,16,17].

To the best of our knowledge, studies on the anti-osteoporotic effects of *C. myrrha* and its constituents are scarce. Considering the effect of estrogen-loss on bone health in postmenopausal women, we hypothesized that *C. myrrha* resin may be a potential therapeutic to treat osteoporosis in this group. Therefore, we investigated whether the water extract of *C. myrrha* resin (WCM) can protect against ovariectomy-induced bone loss in mice. Subsequently, anti-osteoclastogenesis and phytochemical profiling of WCM and its polysaccharide (WCM-PE) were evaluated to elucidate the molecular mechanisms underlying our in vivo findings.

## 2. Results

### 2.1. WCM Protected Estrogen Deprivation-Induced Bone Loss in Mice

**Figure 1:**
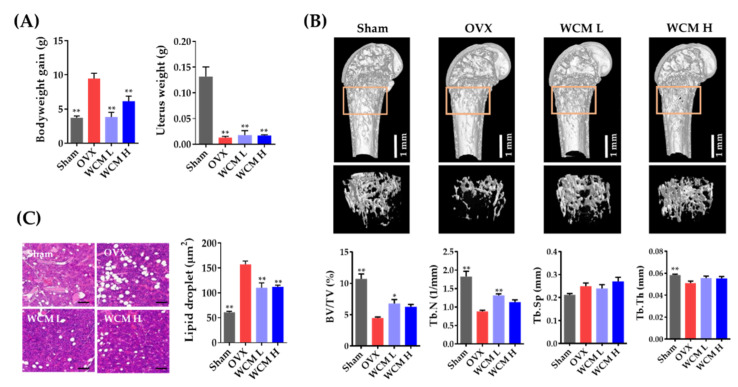
Water extract of *Commiphora myrrha* resin (WCM) alleviates estrogen deprivation-induced bone loss (*n* = 5). (**A**) Alterations in body weight and relative uterus weight in the study groups. (**B**) Three-dimensional micro-CT images and morphometric parameters in micro-CT analysis of the femurs (scale bar, 1 mm). (**C**) Accumulation of lipid droplets in the bone marrow of distal femur detected by hematoxylin and eosin staining (scale bar, 100 µm). Data are represented as mean ± SEM and analyzed by one-way analysis of variance with Dunnett’s post hoc test. * *p* < 0.05 versus OVX, ** *p* < 0.01 versus OVX. Sham, sham-operated/vehicle; OVX, OVX/vehicle; WCM L, OVX/WCM treatment (200 mg/kg/day); WCM H, OVX/WCM treatment (500 mg/kg). BV/TV, bone volume per tissue volume; OVX, ovariectomized mice; H, high-dose; L, low-dose; Tb.N, trabecular number; Tb.Th, trabecular thickness; Tb.Sp, trabecular separation.

Firstly, we investigated the in vivo anti-osteoporotic effects of WCM (200 and 500 mg/kg/day) on estrogen deprivation-induced bone loss in mice. As shown in Figure 1A, OVX mice showed a significant increase in body weight compared to the sham-operated group, whereas the uterine weight was markedly reduced in the OVX group. WCM treatment significantly decreased the apparent increase in body weight of OVX mice, with no statistical change in uterine weight. These results indicate that osteoporosis-related symptoms due to surgical menopause were successfully induced.

In micro-computed tomography (micro-CT) analysis, OVX mice showed thin and small trabecular bones of distal femurs, whereas oral administration of WCM (200 or 500 mg/kg) resulted in more compact and thicker trabeculae than those of the OVX mice (Figure 1B). Consistent with the trabecular microarchitecture, OVX induced a marked decrease in BV/TV, Tb.N and Tb.Th (Figure 1B). Remarkably, WCM treatment significantly restored the diminished BV/TV, Tb.Th and Tb.N levels to normal as compared to the untreated OVX mice. Moreover, histological examination of the bone marrow showed that WCM treatment markedly reduced the accumulation of lipid droplets (Figure 1C). These data suggest that WCM directly alleviates the disruption of bone microarchitecture regardless of the phytoestrogenic effect due to the lack of WCM-related improvement in uterine hypotrophy.

### 2.2. Phytochemical Profile of WCM and WCM-PE

**Figure 2:**
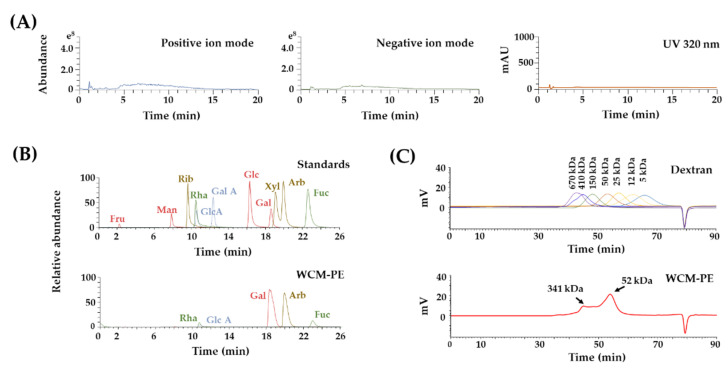
Phytochemical profile of the water extract of *Commiphora myrrha* resin (WCM) and its polysaccharides (WCM-PE). (**A**) Base peak and ultraviolet chromatogram of WCM. (**B**) Monosaccharide composition of WCM-PE. (**C**) High-performance size-exclusion chromatography refractive index (HPSEC-RI) chromatogram of WCM-PE. Fru, fructose; Man, mannose; Rib, ribose; Rha, rhamnose; Glc A, glucuronic acid; Gal A, galacturonic acid; Glc, glucose; Gal, galactose; Xyl, xylose; Arb, arabinose; Fuc, fucose.

High-resolution and accurate mass measurements of plant-derived products have several advantages, including the rapid exploration of noteworthy phytochemicals in a complex matrix and the identification of commercially unavailable compounds. To clarify the biological properties of WCM, we evaluated its chemical characteristics using a UHPLC-quadrupole-orbitrap system. Unexpectedly, few noticeable phytochemicals were detected (Figure 2A). Therefore, we focused on the polysaccharide of WCM as a potential bioactive macromolecule.

To characterize the polysaccharide composition of WCM, we measured its sugar content, mono-saccharide composition and molecular weight of WCM-PE. WCM-PE was rich in sugars and comprised 67.8% neutral sugar and 34.6% uronic acid (Table 1). Upon closer inspection using UHPLC-MS/MS and pre-column derivatization, we found that WCM-PE was mainly composed of galactose (65.6 mol%), arabinose (29.8 mol%), glucoronic acid (2.86 mol%), rhamnose (0.96 mol%) and fucose (0.78 mol%) (Table 1 and Figure 2B). The molecular weight profile analysis using HPSEC revealed two major peaks (341 and 52 kDa) (Figure 2C). Consistent with our findings, the crude gum from the alcohol insoluble ingredients of myrrh reportedly contains contain 64% carbohydrates, composed of galactose, arabinose and glucuronic acid [14,18].

### 2.3. WCM and WCM-PE Inhibited Osteoclastogenesis

**Figure 3:**
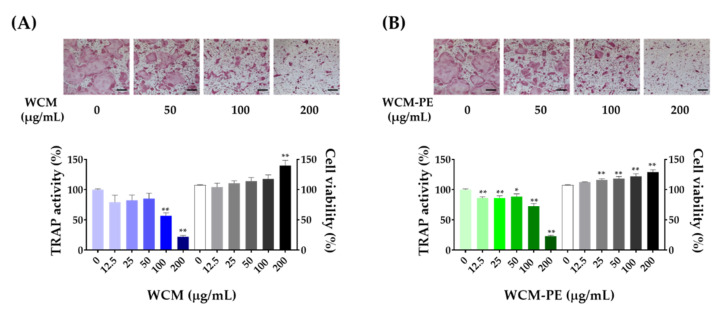
*Commiphora myrrha* exhibits an anti-osteoclastogenic effect on RANKL-stimulated BMMs regardless of cytotoxicity. (**A**) Water extract of *C. myrrha* resin (WCM), (**B**) Polysaccharide extract of WCM (WCM-PE). Inhibitory effect of WCM and WCM-PE (0–200 μg/mL) on osteoclast formation under tartrate-resistant acid phosphatase (TRAP) staining. Results are presented as mean ± SEM and analyzed by one-way analysis of variance with Dunnett’s post hoc test. * *p* < 0.05 versus control. ** *p* < 0.01 versus control. Scale bar, 100 μm.

Considering the beneficial effects of WCM on bone loss in vivo, we next examined whether WCM and its major constituents (WCM-PE) can modulate osteoclastogenesis using bone marrow-derived macrophages (BMDMs). In the presence of M-CSF and RANKL, we treated the cells with WCM and WCM-PE (12.5–200 μg/mL) and evaluated the formation of TRAP-positive multinucleated cells and TRAP activity; RANKL treatment alone fully differentiated TRAP-positive multinucleated cells (Figure 3). Both WCM and WCM-PE treatments dose-dependently reduced the formation and TRAP activity of osteoclasts compared to those in the control group. Moreover, there was no WCM- or WCM-PE-related cytotoxicity at any of the tested doses, excluding the possibility of osteoclastogenesis inhibition due to cytotoxicity. Thus, these findings indicate that WCM and WCM-PE could interrupt RANKL-induced osteoclastogenesis.

### 2.4. WCM and WCM-PE Suppressed Key Transcription Factors and Osteoclast-Specific Genes

**Figure 4:**
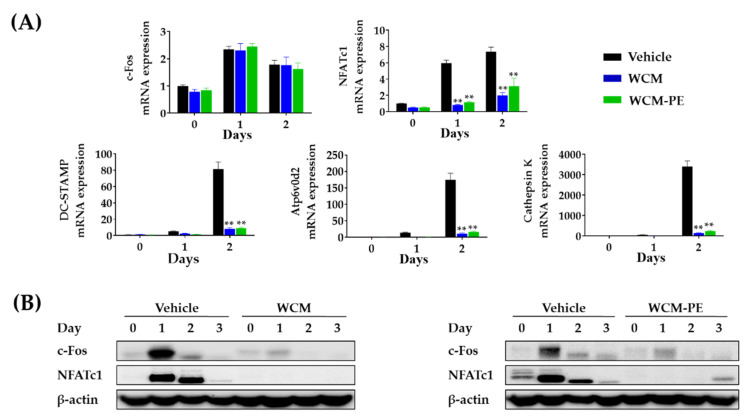
Molecular mechanism underlying the water extract of *Commiphora myrrha* resin (WCM)-and its polysaccharides (WCM-PE)-related modulation in osteoclastogenesis of bone marrow-derived macrophages (BMDMs). (**A**) Inhibitory effects of WCM and WCM-PE on mRNA expression of key transcription factors and osteoclast-specific genes implicated in cell fusion and maturation. (**B**) Inhibitory effects of WCM and WCM-PE on protein expression of c-Fos and NFATc1. β-actin was used as control. Quantitative real-time PCR data are presented as mean ± SEM and analyzed by two-way ANOVA with Bonferroni test., ** *p* < 0.01 versus vehicle control.

Based on the potent inhibitory effect of WCM and WCM-PE on the RANKL-induced osteoclast precursors, we elucidated their effects on mRNA and/or protein expression of key transcription factors and osteoclast-specific genes. Notably, both WCM and WCM-PE treatment (200 μg/mL) reduced the expression of NFATc1 and c-Fos during osteoclast differentiation at the mRNA and protein levels (Figure 4). Furthermore, the downregulation of these key transcription factors by WCM and WCM-PE inhibited the transcriptional expression of DC-STAMP, Atp6v0d2 and cathepsin K (Figure 4A). These results imply that WCM and WCM-PE may suppress early stage osteoclastogenesis by downregulating osteoclast-specific transcription factors.

## 3. Discussion

The last several decades have seen a growing interest in the treatment and management of osteoporosis with herbal medicine-derived products because they not only have fewer undesired effects but are also more adaptable for long-term use in comparison with chemically synthesized anti-osteoporotic agents [7]. Accumulating scientific evidence suggests that anti-osteoporotic natural products and nutraceuticals can be used as cost-effective alternatives to commercial medicines. In this context, we determined the anti-osteoporotic effects of WCM on estrogen deprivation-induced bone loss and elucidated the precise mechanism underlying this phenomenon using WCM and its major bioactive constituent (WCM-PE). 

OVX animal, with surgically removed ovaries, is an excellent experimental model for evaluating ovarian function and menopause-associated disorders, including osteoporosis. In particular, a mouse model is often used to elucidate the modulatory effects of ovarian hormones or to evaluate the anti-osteoporotic properties of candidate agents by evaluating their ability to improve body weight, alleviate apparent uterine hypotrophy and prevent rapid bone loss in postmenopausal osteoporosis [19]. In addition, a high-fat diet supplementation can increase the susceptibility of OVX mice to bone loss [20,21]. Estrogen deficiency by OVX leads to a gradual decrease in bone volume and bone mineral density because of their close association with bone remodeling and homeostasis [19,20]. Ovariectomy in mice can induce significant trabecular bone loss over several weeks and the periodic alteration of bone morphology has been well characterized using micro-CT [22,23,24]. Three-dimensional analysis by micro-CT can more accurately determine the bone microarchitecture and precisely compare the alterations in bone structure, thereby improving the diagnosis and evaluation of osteoporosis [23]. In this study, the oral administration of WCM attenuated OVX-induced bone loss and the accumulation of bone marrow lipid droplet. These results indicate that WCM is sufficient to be a therapeutic candidate for estrogen deficiency-induced osteoporosis.

Osteoclasts that are highly specialized in resorbing bone matrix in bone remodeling are derived from the monocyte/macrophage lineage of the bone marrow [4]. Pathophysiological alterations in the differentiation or function of osteoclasts, as the principal bone-resorbing cells in the body, can cause detrimental effects on the skeletal structure [25]. In osteoclast differentiation, NFATc1 and c-Fos are considered indispensable master regulators [26,27]. In the early stage, the downregulation of any of these proteins prevents osteoclast formation [28]. RANKL stimulation leads to an increase in NFATc1 expression through c-Fos and its autoamplification activates the transcription of osteoclast-specific genes (e.g., Atp6v0d2, DC-STAMP and cathepsin K) involved in cell fusion and bone matrix degradation [29,30]. In the present study, WCM and WCM-PE had shown the inhibition of osteoclastogenesis and the expression of osteoclast-specific genes via the downregulation of c-Fos and NFATc1 expression. These data suggest that the modulatory effects of WCM and its polysaccharide on osteoclast differentiation could be partially contributed to the anti-osteoporotic effects of WCM in OVX mice. 

Plant-derived polysaccharides, as essential biological macromolecules, have drawn much attention owing to their significant bioactive, biocompatible and biodegradable properties [31]. In modern pharmacological studies of traditional oriental medicines, the polysaccharides of herbal medicines have been confirmed as major bioactive components, which are responsible for various pharmacological activities such as antioxidant, antitumor, anti-hyperglycemic and immune-stimulatory activities [32]. Especially, several recent studies have demonstrated that bioactive polysaccharides isolated from different plants have potent anti-osteoporotic effects in vivo [33,34,35]. In our study, *C. myrrha* resin-derived polysaccharides (i.e., WCM-PE) markedly inhibited RANKL-induced osteoclastogenesis, which was similar to that of WCM. In this regards, WCM-PE could be an excellent candidate to manage bone health and osteoporosis. However, further studies are required to clarify in vivo efficacy, bioavailability and structural characterization of WCM-PE.

## 4. Materials and Methods

### 4.1. Chemicals and Reagents

Water, acetonitrile, alpha-modified minimum essential medium Eagle (α-MEM) and fetal bovine serum (FBS) were purchased from Thermo Fisher Scientific Inc. (Rockford, IL, USA). Arabinose, fucose, galactose, galacturonic acid, glucose, glucuronic acid, mannose, rhamnose, ribose, xylose, dextran series and p-nitrophenyl phosphate (pNPP) were purchased from Sigma-Aldrich (St. Louis, MO, USA). Specific antibodies against NFATc1, c-Fos and β-actin as well as secondary antibodies were obtained from Santa Cruz Biotechnology (Santa Cruz, CA, USA). 

### 4.2. Preparation of WCM and WCM-PE

*C. myrrha* resin used in this study was purchased from a herb good manufacturing practice (hGMP) manufacturer (OMniherb, Seoul, Republic of Korea). A voucher specimen (#KW-7) of raw material was deposited in the herbarium of the Herbal Medicine Research Division. The origin of *C. myrrha* resin was authenticated by Dr. J. So, National Development Institute of Korean Medicine (NIKOM, Gyeongsan, Republic of Korea). Dried *C. myrrha* resin (0.5 kg) was extracted with distilled water (3.5 L) under reflux for 3 h and then dried using a vacuum freeze dryer (Ilsinbiobase, Dongduchun, Republic of Korea). Cold ethanol was added to it at a final concentration of 80% (*v/v*) and the solution was maintained at −20 °C for 12 h. The precipitate was obtained, dissolved in water, deproteined using the Sevage method and ultrafiltered using a Vivaspin 20 (Sartorius, Goettingen, Germany; filter cut off 10 kDa). The high-molecular-weight polysaccharide fraction of the WCM was lyophilized (yield, 58.4%). The WCM and WCM-PE powders were stored at −20 °C until further use.

### 4.3. Chemical Profiling of WCM and WCM-PE

To identify the phytochemical profile of WCM using ultra-high-performance liquid chromatography-tandem mass spectrometry (UHPLC-MS/MS), a Dionex UltiMate 3000 system equipped with a Thermo Q-Exactive mass spectrometer was used and data acquisition was performed using Xcalibur v.4.2. software (Thermo Fisher Scientific, San Jose, CA, USA). Chromatographic separation was performed using a C18 column (Ac-quity BEH; 100 × 2.1 mm, 1.7 μm) with acetonitrile and 0.1% formic acid in water, ac-cording to a previously reported method [36]. 

The contents of total sugar and uronic acid in WCM-PE were measured by the phenol-sulfuric acid method [37] and carbazole-H_2_SO_4_ method [38], respectively, using galactose and galacturonic acid as the respective standards. The thiobarbituric acid method [39] was used to determine the content of 2-keto-3-deoxy-mannooctanoic acid in WCM-PE. The protein content was analyzed using the Bradford method with protein assay dye (Bio-Rad laboratories, Hercules, CA, USA) and bovine serum albumin. The sugar composition of WCM-PE was determined by UHPLC-MS/MS, according to a previously reported method with some modifications [40]. Reference standards and WCM-PE hydrolysate generated by trifluoroacetic acid were derivatized with 1-phenyl-3-methyl-5-pyrazolone (PMP). The monosaccharides in the acid hydrolysates were separated using an Acquity BEH C_18_ column (150 × 2.1 mm, 1.7 μm) with acetonitrile and 25 mM ammonium acetate in water (pH 8.0, adjusted with ammonia). Eleven reference standards were used to confirm the monosaccharides based on their retention times and fragment patterns in the mass spectra. The molecular weight pattern of WCM-PE was measured using a high-performance size-exclusion chromatography system (HPSEC) with a refractive index detector (Shimadzu Nexera X2, Kyoto, Japan). The serially connected Asahi-pak GS-620, GS-520 and GS-320 columns (0.76 × 30 cm, each; Showa Denko Co., Tokyo, Japan) were eluted with 50 mM ammonium formate (pH 5.5, adjusted with formic acid) at a 0.40 mL/min flow rate. The injection volume was 25 μL. A standard curve was constructed using a dextran standard set (D-667, 410, 147, 49, 24, 11.6, 5.2 kDa) with the following equation: Log Mw = −0.0893 RT + 9.5375 (R2 = 0.992).

### 4.4. Animal Study

Female C57BL/6 mice (6-week-old) were obtained from Japan SLC (Shizuoka, Ja-pan). Mice were housed under standard conditions and supplied with water and a standard chow diet ad libitum during the acclimation period of one week. Animals were randomly assigned into four groups (*n* = 5/group) to investigate the effect of estrogen deficiency on bone loss. Under anesthesia with Zoletil 50 (Virbac, Carros, France) and Rumpun (Bayer, Leverkusen, Germany), the mice were either sham-operated (control group) by bilateral dorsal laparotomy or ovariectomized (OVX) through double dorsolateral incisions. The OVX mice were randomly divided into three groups based on treatment: vehicle (negative control), WCM-L (200 mg/kg/day) and WCM-H (500 mg/kg/day). WCM was administered by oral gavage once daily for four weeks. One week after the surgery, the animals were fed high-fat diet (60 kcal %), pro-vided by Research Diet Inc. After four weeks, mice were sacrificed by an overdose of Zoletil and Rumpun and femoral specimens were collected for further analysis. 

For histological examination, formalin-fixed femurs were embedded in paraffin, cut into 5 µm thick sections, stained with hematoxylin and eosin and then observed for lipid accumulation under a light microscope. The microarchitecture of the distal femur was analyzed using the Quantume GX microCT imaging system (PerkinElmer, Inc., Hopkinton, MA, USA) according to a previous report [36]. After reconstructing the scanned images, we determined the bone morphometric parameters, including bone volume/tissue volume (BV/TV), trabecular number (Tb.N), trabecular thickness (Tb.Th) and trabecular separation (Tb.Sp) using the SkyScan software (Bruker, Kontich, Belgium).

### 4.5. Osteoclast Differentiation Assay

BMDMs were prepared and cultured as previously described [41]. BMDMs were seeded in 96-well plates (1 × 10^4^ cells/well) or 6-well plates (1 × 10^5^ cells/well). BMDMs were treated with different concentrations of WCM and WCM-PE (0–200 μg/mL) for 1 h, followed by RANKL treatment to induce osteoclast differentiation and then cultured for three days. Thereafter the cells were fixed and permeabilized and osteoclast formation and maturation were evaluated by TRAP staining, as previously reported [41]. TRAP-positive multinucleated cells derived from BMDMs were identified by microscopic observation. TRAP activity was also deter-mined by using the phosphatase substrate, pNPP. The viability of BMDMs treated with WCM and WCM-PE (0–200 μg/mL) was measured using the CCK-8 assay (Dojindo Mo-lecular Technologies Inc., Tokyo, Japan). 

### 4.6. Real-Time PCR and Western Blotting

Total RNA was extracted from BMDMs using the RNeasy Mini kit (Qiagen, Hilden, Germany) in accordance with the manufacturer’s protocol. Total RNA (1 μg) was incubated with reverse transcriptase, oligo-dT primer and deoxynucleoside triphosphate to prepare cDNA using High Capacity cDNA Reverse Transcription Kit from Applied Biosystems Inc. (ABI, Foster City, CA, USA). The reaction mixture with 1μL of cDNA was prepared using a TaqMan Universal PCR Master Mix and then analyzed by real-time quantitative PCR using an ABI 7500 Real-Time PCR System (ABI, Foster City, MA, USA). Primers for c-Fos (Mm00487425_m1), NFATc1 (Mm00479445_m1), Atp6v0d2 (Mm00656638_m1), DC-STAMP (Mm01168058_m1), cathepsin K (Mm00484036_m1) and 18S ribosomal RNA (rRNA, Hs99999901_s1) were supplied by ABI (Foster City, CA, USA). 18S rRNA, a stable housekeeping (reference) gene was used for normalization.

For western blotting, cell lysates were prepared in RIPA lysis buffer containing protease and phosphatase inhibitor cocktails (Roche Diagnostics, Indianapolis, IL, USA). Protein content was quantified using a BCA kit (Thermo Fisher Scientific Inc., Rockford, IL, USA); protein samples were separated by SDS-PAGE, transferred onto a PVDF membrane and blotted using primary (1:1000 dilution) and secondary (1:2000 dilution) antibodies. Chemiluminescent signals were detected using a ChemiDoc imaging system (Bio-Rad Laboratories, CA, USA).

### 4.7. Statistical Analysis

All data are represented as mean ± standard error of mean (SEM). Differences among groups in animal study or treatments in osteoclast differentiation assay were assessed by one-way analysis of variance (ANOVA). If significant difference was indicated, then difference between the two groups or treatments was tested by *Dunnett’s* post-hoc test. *Data from real-time PCR test* were analyzed by two-way ANOVA with treatment (vehicle, WCM and WCM-PE) and exposure time (0, 1 and 2 days) as factors, followed by Bonferroni test for multiple comparisons. *p*-values below 0.05 were considered statistically significant. *p* values of less than 0.05 were considered statistically significant.

## 5. Conclusions

Plant-derived products are natural alternatives to conventional therapies that can efficiently manage bone disorders. To the best of our knowledge, this is the first study to demonstrate the protective effects of myrrh and its polysaccharides (i.e., WCM-PE) on bone health. We found that WCM ameliorated estrogen deprivation-induced bone loss, a hallmark of osteoporosis and the anti-osteoporotic effects of WCM and WCM-PE could be attributed to its potential to suppress osteoclastogenesis by downregulating c-Fos/NFATc1. Therefore, myrrh could be a promising candidate for the management of postmenopausal osteoporosis.

## Figures and Tables

**Table 1:** Chemical composition of the polysaccharide fraction isolated from the water extract of *Commiphora myrrha* resin (WCM-PE).

	WCM-PE
Chemical composition (%)	
Neutral sugar	67.78 ± 1.79
Uronic acid	34.58 ± 0.36
2-keto-3-deoxy-mannooctanoic acid	0.20 ± 0.02
Protein	2.65 ± 0.13
Component sugar (mol%)	
Arabinose	29.84
Fucose	0.78
Galactose	65.56
Rhamnose	0.96
Glucoronic acid	2.86

Mol% was calculated based on the detected total sugar.

## Data Availability

The data presented in this study are available on request from the corresponding author.
